# Mapping Localized Peroxyl Radical Generation on a PEM Fuel Cell Catalyst Using Integrated Scanning Electrochemical Cell Microspectroscopy

**DOI:** 10.3389/fchem.2020.572563

**Published:** 2020-10-21

**Authors:** Joseph Edgecomb, Xiaohong Xie, Yuyan Shao, Patrick Z. El-Khoury, Grant E. Johnson, Venkateshkumar Prabhakaran

**Affiliations:** Pacific Northwest National Laboratory, Richland, WA, United States

**Keywords:** oxygen reduction reaction, fuel cell, peroxide generation, reactive oxygen species, *in situ* fluorescence probe

## Abstract

Understanding molecular-level transformations resulting from electrochemical reactions is important in designing efficient and reliable energy technologies. In this work, a novel integrated scanning electrochemical cell microspectroscopy (iSECCMS) capability is developed by combining a high spatial resolution electrochemical scanning probe with *in situ* fluorescence spectroscopy. Using 6-carboxyfluorescein as a fluorescent probe, the iSECCMS platform is employed to measure the effect of the detrimental generation of reactive oxygen species (ROS) formed at the active sites of oxygen reduction reaction (ORR) catalysts. Carbon-supported tantalum-doped titanium oxide (TaTiO_x_) catalysts, a potential Pt-group-metal-free (PGM-free) cathode material explored for low temperature polymer electrolyte fuel cells (PEFCs), is used as a representative model ORR system, where generation of intermediate H_2_O_2_ instead of fully oxidized H_2_O is a major concern. We establish that the iSECCMS platform provides a novel and versatile capability for spatially resolved mapping of *in situ* ROS generation and activity during the kinetically-limited ORR and may, therefore, aid the future characterization and development of high-performance PGM-free PEFC cathodes.

## Introduction

Increasing global demand for energy has necessitated the development of highly efficient energy conversion and storage technologies (Li et al., 2016). The rational development of efficient energy systems requires an in-depth understanding of the intrinsic electrochemical activity of the operating interface, which usually features substantial complexity and surface heterogeneity (Zhu et al., 2017; Zhang et al., 2018). While traditional electrochemical techniques using direct and alternating current [e.g., cyclic voltammetry (CV), electrochemical impedance spectroscopy (EIS)] have been established for macromolecular measurements to determine bulk-level activity, these techniques generally do not monitor activity and chemical changes directly at local active sites on the electrode. The ability to measure and control individual electrochemical reactions is important in establishing a molecular-level understanding of the surface chemistry of isolated sites on electrode–electrolyte interfaces (EEIs). For example, the activity and degradation of electroactive catalysts vary substantially based on the initial distribution and subsequent aggregation of catalyst particles on electrodes during operation (Sui et al., 2017). Furthermore, the structure of the pores through which reactant molecules diffuse at the electrode surface is dependent on the spatial location and distribution of the electrocatalyst (Hess et al., 2011). Spatially localized electrochemical methods coupled to structural characterization tools are required to understand these spatially varying properties of operating electrochemical interfaces.

*In situ* site-specific scanning electrochemical techniques have been indispensable in understanding the local surface chemistry of electrodes used for heterogeneous energy conversion and storage (Wu et al., 2008; Sannomiya et al., 2010; Byers et al., 2014a; Chirea et al., 2014; Wang and Tao, 2014; Brasiliense et al., 2016; Wang et al., 2016). In particular, newly developed scanning electrochemical methods, such as pipette-based scanning electrochemical cell microscopy (SECCM) (McKelvey et al., 2010; Byers et al., 2014b; Daviddi et al., 2019; Wordsworth et al., 2020) and conventional tip-based scanning electrochemical microscopy (SECM) (Kai et al., 2018), may be used to map local activity across the surface of electrodes. The former technique has been shown to be especially useful in measuring the activity of so-called “hot spots” on electrode surfaces, which occur when the heterogeneous distribution of electrical double layers on charged catalytic surfaces overlap, resulting in regions of increased reactant adsorption and electroactivity (Holt et al., 2004). These spatially localized scanning methods can be further enhanced when coupled with Raman (Ramesha and Sampath, 2009), Fourier transform infrared (FTIR) (Fang et al., 2009; Su et al., 2018), and other spectroscopic methods (Wang et al., 2016), thereby providing additional information about chemical changes occurring during electrochemical measurements. Therefore, the integration of spectroscopy with existing electrochemical scanning techniques may yield a transformative approach to characterizing complex electrochemical interfaces (Bentley et al., 2017; Baker, 2018).

In fuel cells, a spatially resolved single-entity electrochemical technique can be used to study the molecular-level catalytic activity of oxygen reduction reaction (ORR) catalysts. The effects of the coordinating environment, particle distribution, and crystal structure on the performance of Pt-based ORR catalysts have been studied for decades (Kinoshita, 1992). Characterization of Pt catalysts in the mid-2000s suggested that particle size is a strong determinant of ORR activity (Mayrhofer et al., 2005). However, more recent experiments indicate that the spatial distribution of particles on the electrode surface is also strongly correlated to catalyst performance. For example, in 2013, Arenz and coworkers showed that the specific activity (measured with a bulk electrochemical cell) of Pt ORR catalysts is dependent on the distance between nanoparticles, rather than particle size (Nesselberger et al., 2013). In their study, the edge-to-edge distance between particles was calculated assuming an even distribution of catalyst on the electrode surface, with SEM imaging indicating that the Pt catalyst particles were immobile on the electrode surface. In cases when the catalyst distribution is heterogeneous and dynamic, *in situ* spatially resolved techniques may be required. Characterization of the ORR performance is further complicated by the generation of reactive oxygen species (ROSs), namely, hydroxyl and hydroperoxyl radicals formed by decomposition of the hydrogen peroxide (H_2_O_2_) intermediate on the carbon support of the Pt catalysts (Trogadas et al., 2011; Wang et al., 2019; Trogadas and Coppens, 2020). ROSs attack the polymer binding holding the catalysts to the electrode, resulting in substantial particle aggregation and variation in edge-to-edge distances between particles. The spatial resolution provided by single-entity electrochemical techniques such as SECM and SECCM allows monitoring of local particle aggregation and the active site distribution—properties that are essential for characterizing and understanding ORR activity.

Current interest in ORR catalysts is driven in part by their application in polymer electrolyte fuel cells (PEFCs). These fuel cells operate through the reduction and oxidation of oxygen and hydrogen at the cathode and anode, respectively. Generally, a fluorinated polymer such as Nafion® and Flemion® is utilized as the electrolyte. PEFCs have been identified as a promising source of renewable energy for future transportation applications, but are currently limited in their use by the prohibitively high costs and performance degradation of the Pt-group metal (PGM)-based cathodes required to promote the ORR (Litster and McLean, 2004; Hess et al., 2011; Debe, 2012; Epting and Litster, 2012; Houchins et al., 2012; Coleman and Co, 2015; Hu et al., 2019). Various metal oxides and alternative organometallics have been investigated as low Pt and PGM-free catalysts for the ORR (Ishihara et al., 2008; Coleman and Co, 2014; Du et al., 2020). Perhaps the most active PGM-free ORR catalysts are ligated iron species formed by sintering of organometallic precursors with argon or nitrogen (Lefèvre et al., 2009; Sun et al., 2017; Zhang et al., 2017; Li et al., 2019). Unfortunately, leached iron from ligated iron catalysts promotes decomposition of intermediate hydrogen peroxide, formed during the ORR, into ROS (Wang et al., 2019; Du et al., 2020). In addition to the deleterious effects of ROS generation, other components of PEFCs are especially sensitive to radical formation. Specifically, the polymer membrane will degrade in the presence of ROS, eventually forming pinholes that lead to complete cell failure (Mittal et al., 2006; Coms, 2008; Gubler et al., 2011). Substantial ROS generation persists even in iron-free and PGM-free ORR catalysts, emphasizing the importance of this reaction (Wang et al., 2019; Du et al., 2020).

Understanding the localized generation of ROS on electrodes with immobilized ORR catalysts is the key to enabling the rational design of efficient and stable cathodes for PEFCs. Various methods of monitoring the generation of ROS during the ORR on catalyst layers have been reported in the literature. For example, electron paramagnetic resonance (EPR) spectroscopy has been used to measure ROS generation in *ex situ* systems, but this method lacks the spatial resolution necessary to understand the role of electrode heterogeneity (Panchenko et al., 2004a,b; Bosnjakovic et al., 2007; Fang et al., 2009). Fluorescence spectroscopy methods were first reported by Nosaka et al. as an alternative way to monitor ROS generation with *ex situ* measurements (Murakami et al., 2006). A suitable fluorescent probe sensitive to ROS, such as 6-carboxyfluorescein dye (6CFL), is added to the catalyst layer. In the presence of ROS, the fluorescence of the dye is quenched, providing a real time method to monitor ROS generation (Prabhakaran et al., 2012). However, because the lifetime of radicals is very short (~10^−9^ s), developing *in situ* detection methods that can be used in an operating environment remains challenging.

Recently, *in situ* fluorescence-based methods were incorporated into membrane electrode assemblies (MEA) to correlate the bulk generation of ROS with operating fuel cell parameters (i.e., temperature, relative humidity) and measure the efficacy of ROS scavengers (e.g., CeO_x_) used to mitigate the negative effects of ROS/H_2_O_2_ generation (Prabhakaran et al., 2013; Prabhakaran and Ramani, 2014). While these methods have been useful to establish the correlation between MEA activity and ROS generation on the bulk scale, it remains important to characterize ROS generation at the catalyst layer. The heterogeneity in size, shape, and distribution of the ORR catalyst particles and the presence of aggregated or depleted regions may affect the ORR and generation of ROS. By integrating fluorescence techniques with spatially resolved SECCM, the effect of these nanostructural features may be better understood for ORR catalysts.

**Figure 1:**
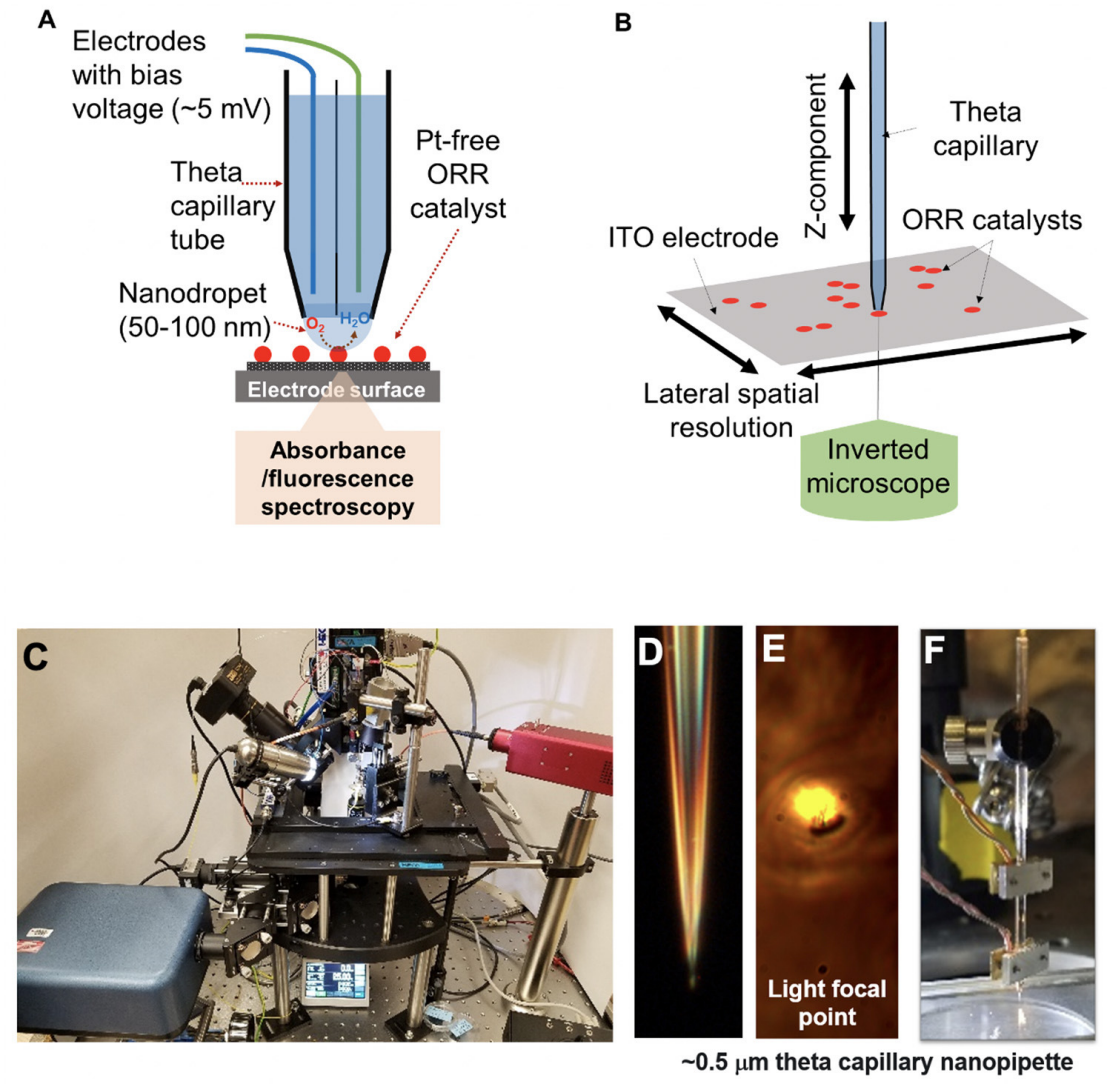
**(A)** Schematic diagram of the integrated scanning electrochemical cell microspectroscopy (iSECCMS) platform that combines scanning electrochemical cell microscopy (SECCM) with fluorescence spectroscopy. **(B)** The capillary tip of the iSECCMS platform can be moved in the XYZ directions, which enables the operator to accurately position the measurement location. **(C)** Photograph of the iSECCMS platform. **(D)** Microscope image of a pulled theta capillary used in the platform. **(E)** The focal region of light from the inverted microscope placed under the working electrode. **(F)** The theta capillary mounted with a shear-force assembly to control the position of the tip in the Z-direction while landing it on the electrode surface during measurements.

Herein, we report the development of integrated scanning electrochemical cell microspectroscopy (iSECCMS) to investigate the origin of the degradation of the catalytic activity of nitrogen-doped tantalum–titanium oxide supported on carbon (N-TaTiO_x_/C), a representative model system for PGM-free ORR catalysts (see Figure 1). Rotating ring disk electrode (RRDE) experiments reveal substantial generation of H_2_O_2_ as an intermediate during the ORR on TaTiO_x_/C, which is the primary explanation for the lower ORR activity of this material compared to PGM-based ORR catalysts. N-TaTiOx/C was chosen as a model catalyst to test our iSECCMS platform because: (i) TaTiO_x_ was previously demonstrated to be a durable catalyst support for PGM-based ORR catalysts (Kumar and Ramani, 2013); (ii) N-doping on TaTiOx/C is proposed to create ORR active sites with a more durable ORR catalyst. Therefore, there is future potential to use this catalyst as PGM-free electrodes. We employ our iSECCMS platform to characterize the ORR on a catalyst surface layer made of N-TaTiO_x_/C immobilized with Nafion®/6CFL as a fluorescent probe to monitor ROS production. The measured kinetic activity and reduction potential of the ORR using the iSECCMS platform is in agreement with the *ex situ* RRDE measurements. Increased decay in the fluorescence of 6CFL corresponding to the generation of ROS/H_2_O_2_ was observed on N-TaTiO_x_/C in the presence of O_2_ compared to similar measurements performed in Ar. The findings reported herein illustrate a unique opportunity to evaluate ORR activity and ROS generation on ORR catalyst layers at the sub μm level.

## Methods

### Chemicals

Tantalum (V) ethoxide [Ta(OEt)_5_]−99.98%, titanium (IV) isopropoxide [Ti(OiPr)_4_]−97%, Nafion® perflorinated resin−5 wt%, potassium ferricyanide (III) powder [K_3_Fe(CN)_6_]−99%, potassium chloride (KCl)−99%, 5(6)-carboxyfluorescein (6CFL)−95%, and dimethylformamide (DMF)-99.8% were all purchased from Sigma Aldrich and used as obtained. Sulfuric acid (H_2_SO_4_) was obtained from Fluka Analytical. Ketjen Black (KB) Carbon (EC-600JD) was purchased from Akzo Nobel Polymer Chemicals LLC, Chicago IL, United States.

### Synthesis of N-Doped TaTiO_x_/C

The synthesis procedure for the N-TaTiO_x_/C catalysts was adopted from the literature (Kumar and Ramani, 2013). Tantalum ethoxide and titanium isopropoxide were added in a 1:4 ratio to 20 ml of ethanol. Approximately 5 ml of water was added to the stirred solution until it turned white. The solution was left to stir for 10 min and vacuum filtered. The precipitate was left under vacuum at 70°C overnight to dry. The next day, an equal amount of the catalyst was added to KB carbon in a 1:1 ratio along with 15 ml of water and stirred for 1 h. The solution was vacuum filtered and left at 60°C under vacuum overnight. For N-doping, ~0.1 g of each catalyst was added to ~1.4 g of urea and mixed. The resulting powder was annealed at 850°C for 3 h under argon.

### Preparation of Catalyst Ink and Electrode Fabrication

For *ex situ* measurements using the RRDE three-electrode cell, 10 mg of the ORR catalyst was added to 1990 μL of a (70/30)% isopropanol/water mixture and 10 μL of Nafion® solution. The solution was sonicated (VWR Model 150t) for 30 min and then vortexed at 2,500 rpm for 2 min. Approximately 50 μL of the catalyst ink was drop cast onto a polished glassy carbon (GC) electrode present in the disk region of the RRDE and dried in ambient conditions.

For *in situ* measurements using iSECCMs, a 50% solution of 8.3 mM 6CFL (in DMF) was prepared with Nafion® and used to establish a baseline. For ORR measurements, ~1 mg of catalyst was added to the 6CFL/Nafion® solution and drop cast onto indium-tin oxide (ITO)-coated glass slides (MTI).

### *Ex situ* Ferri/Ferrocyanide Baseline Measurements

The ferri/ferrocyanide redox couple was used as a baseline measurement to establish the reliability of the electrochemical part of the iSECCMS platform. The redox activity of ferri/ferrocyanide was measured *ex situ* using a custom-built Teflon three-electrode bulk cell and a potentiostat (VersaStat 4, Princeton Applied Research). Potentials were recorded in reference to a Ag/AgCl (saturated KCl) electrode purchased from Basi (RK 450, West Lafayette, IN, United States). A Pt wire (0.25 mm diameter, Alfa Aesar) was used as a counter electrode. Polished Sigradur GC slides, 10 × 10 mm^2^, were used as working electrodes. Between each measurement, the cell components were cleaned by sonication in water/isopropanol.

### *Ex situ* RRDE Measurements on N-TaTiO_x_/C

The *ex situ* RRDE experiments to measure the intrinsic ORR activity of N-TaTiO_x_/C were performed using a conventional RRDE electrode setup purchased from Pine Instrument (Grove City, PA, United States) and a potentiostat (CH Instruments). Graphite was used as a counter electrode and a reversible hydrogen electrode (RHE) was used as a reference electrode.

### *In situ* Fluorescence Measurements With the iSECCMS Platform

The electrochemical scanning probe, purchased from HEKA (EPC 10 USB double amplifier), was modified to enable integrated real-time SECCM and UV-Vis fluorescence spectroscopy measurements (see Figures 1A–C). Two-chamber theta glass capillaries (borosilicate theta TG150-4, Warner Instruments) were pulled using a laser capillary puller (Sutter Instruments, Co. P-2000) to a tip diameter of 0.5–20 μm (Figures 1D,E). A 0.5-μm tip was used in the initial baseline ferri/ferrocyanide experiment. For ORR measurements, a slightly larger capillary tip diameter of 0.7 μm was used to allow sufficient oxygen saturation of the electrolyte solution. The spatial resolution of the measured region is defined by the tip diameter of the theta capillary. The HEKA instrument's shear-force assembly was mounted on the theta glass capillary, as shown in Figure 1F, to accurately position the capillary tip on the catalyst layer/ITO-support and establish stable contact between the capillary droplet and the catalyst layer (Figure 1F). Electrolyte, counter and reference electrodes were added to each compartment of the theta capillary depending on the experiment. For the ferri/ferrocyanide experiment, 0.01 M aqueous KCl was used as the electrolyte and Ag and Pt wires were used as the reference and counter electrodes, respectively. For the ORR measurements, 0.1 M H_2_SO_4_ purged alternatively with Ar or O_2_ was used as the electrolyte and Ag and W wires were used as reference and counter electrodes, respectively. The assembled theta capillary was mounted on the iSECCMS platform as shown in Figure 1C. The catalyst layer/ITO acts as the working electrode. During ORR measurements, the entire iSECCMS platform is covered with a plastic enclosure to prevent inflow of other gases. Oxygen was purged locally where the capillary tip approaches the catalyst layer/ITO-support. In the *in situ* ORR experiments performed on iSECCMS, the potentials of voltammetry, measured using an Ag reference electrode, were adjusted to the RHE scale by adding 0.20 V, assuming it acts as a conventional Ag reference electrode.

For optical measurements using the 6CFL fluorescent probe on iSECCMS, a 495-nm compact continuous wave diode laser (Thor Labs, Model CLD1011LP) was aligned with the optically transparent capillary tip using the objective lens of an inverted microscope. The objective lens (20 × air objective, NA = 0.4) was used to focus the excitation laser and collect the emission signal from the focal volume. The transparent ITO electrode allowed an inverted microscope setup to be used in this platform. Emission data was collected with a compact spectrometer (Thor Labs, CCS200). Each *in situ* fluorescence measurement was repeated three times, and error bars were added to the respective plots. Prior to *in situ* measurement, UV-Vis fluorescence spectra of a prepared 6CFL/Nafion® solution were obtained in a 1-cm quartz cuvette with a deuterium–tungsten light source (AIS DT2000).

## Results and Discussion

**Figure 2:**
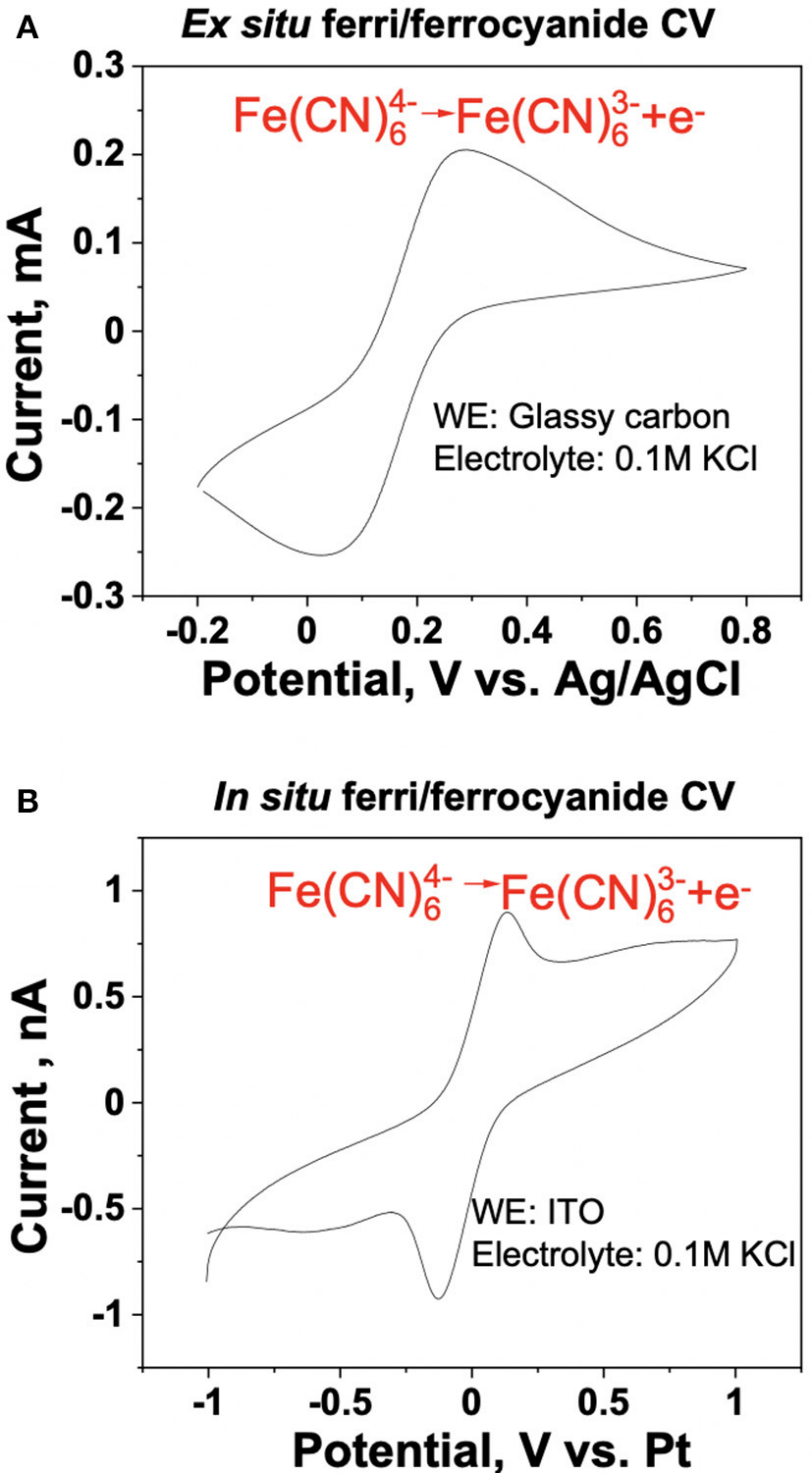
Benchmarking of the iSECCMS platform using the ferri/ferrocyanide redox couple: cyclic voltammogram of ferri/ferrocyanide in **(A)** *ex situ* three-electrode bulk cell measurement of 0.14 mM ferrocyanide in 0.1 M KCl, at 50 mV/s from −0.2 to 0.8 V and **(B)** *in situ* iSECCMS scan of 1 mM ferrocyanide in 1 mM KCl at 50 mV/s from −1 to 1 V.

**Figure 3:**
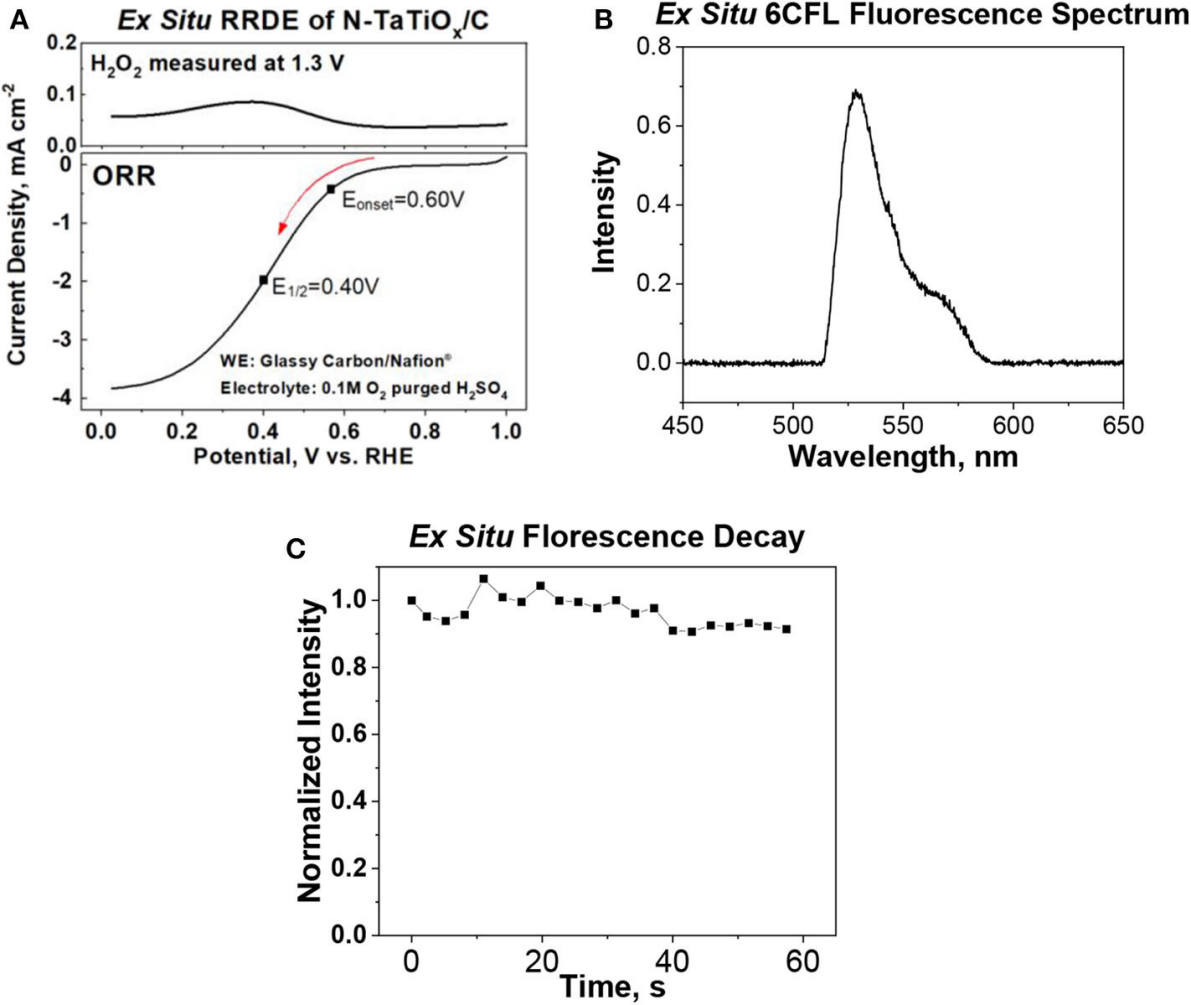
**(A)** *Ex situ* rotating ring disk electrode (RRDE) measurements showing the oxygen reduction reaction (ORR) polarization curve of N-tantalum-doped titanium oxide (TaTiO_x_)/C and H_2_O_2_ generation current measured during the ORR by maintaining the ring potential of the RRDE at 1.3 V (electrolyte: 0.1 M H_2_SO_4_ saturated with O_2_), **(B)** *ex situ* fluorescence emission spectrum of 6-carboxyfluorescein dye (6CFL) in water, and **(C)** *ex situ* fluorescence of 6CFL in water over time.

**Figure 4:**
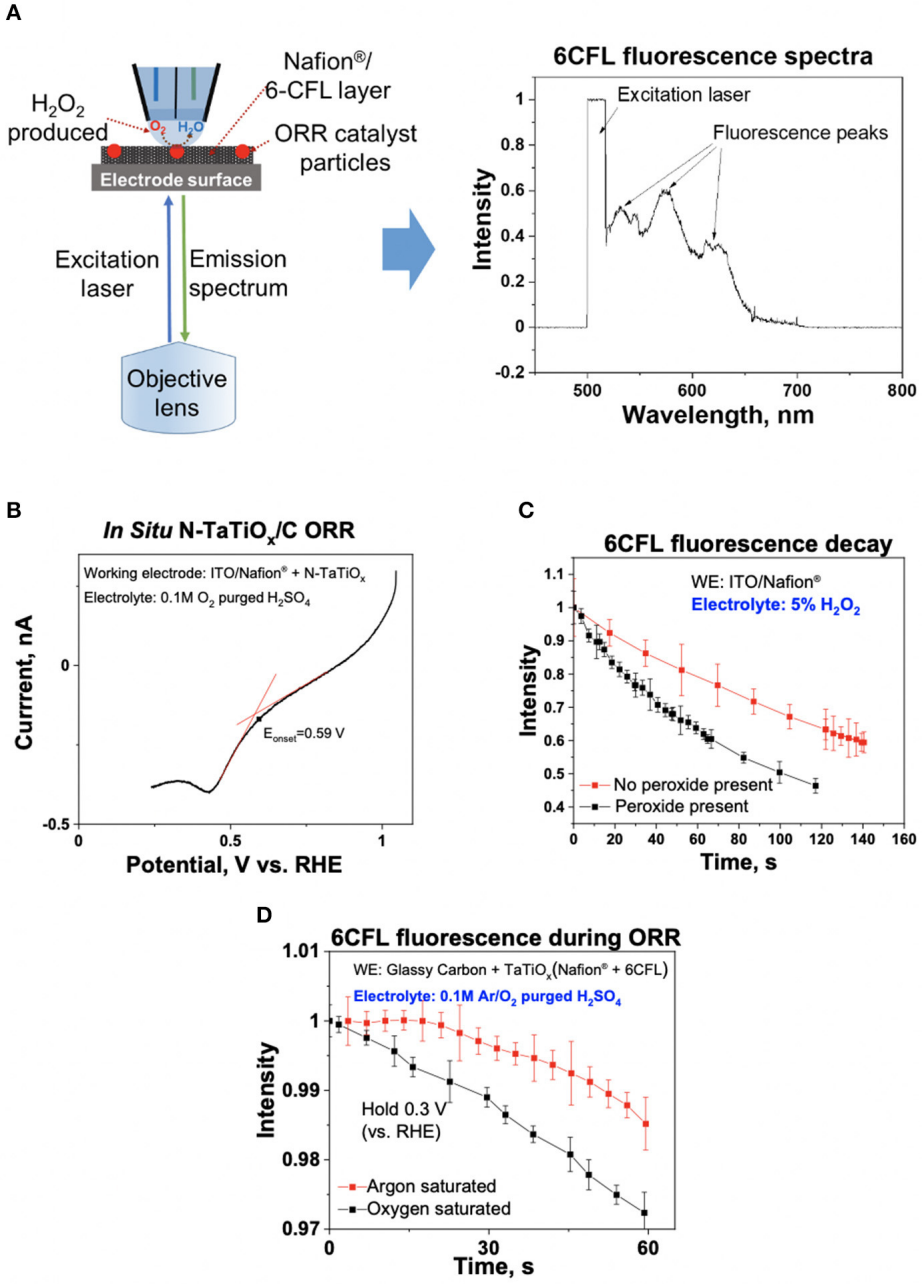
**(A)** Pt-group-metal (PGM)-free oxygen reduction reaction (ORR) catalyst, N-TaTiOx/C, was immobilized with 6CFL and Nafion® on an indium-tin oxide (ITO) electrode, and an inverted microscope was used to monitor the fluorescence spectra of 6CFL. The measured fluorescence spectrum is shown. **(B)** ORR polarization curve of N-TaTiOx/C dispersed on a Nafion® film with oxygen-saturated 0.1 M H_2_SO_4_ electrolyte at a scan rate of 10 mV/s. **(C)** Fluorescence decay of 6CFL with and without 5% H_2_O_2_ added in the electrolyte solution in the theta capillary (no catalyst present on ITO). **(D)** Fluorescence decay of 6CFL measured using Ar and O_2_-saturated 0.1 M H_2_SO_4_ electrolyte with N-TaTiOx/C on ITO. The working electrode potential was maintained at 0.3 V vs. reversible hydrogen electrode (RHE).

*Ex situ* and *in situ* cyclic voltammetry (CV) measurements of the ferri/ferrocyanide redox couple were performed to evaluate and benchmark the reliability of the iSECCMS platform (Figure 2). These measurements showed the characteristic one-electron redox transfer of the ferri/ferrocyanide redox couple, which established the ability of the iSECCMS platform to make reliable electrochemical measurements. Similarly, the ORR activity of N-TaTiO_x_/C was measured with linear sweep voltammetry (LSV) in an *ex situ* RRDE setup and compared to an *in situ* measurement performed with iSECCMS. The LSV of the *ex situ* RRDE showed the onset potential for the ORR at 0.6 V vs. RHE (see Figure 3A). The *in situ* measurement using the theta capillary showed the onset potential for the ORR at 0.59 V vs. RHE (see Figure 4B), which is within the error of the measurements. This result demonstrates the ability of the platform to reliably measure ORR catalytic activity. In addition, the RRDE measurement showed an increase in the H_2_O_2_ generation current, which confirms the partial reduction of oxygen and the possibility of ROS generation on the N-TaTiO_x_/C catalyst layer. Even though we are not able to measure H_2_O_2_ generation directly with the iSECCMS platform, we assert that H_2_O_2_/ROS generation likely occurs on N-TaTiO_x_/C during *in situ* measurement using iSECCMS.

To evaluate the spectroscopy component of the platform, the fluorescence spectrum of 6CFL was measured using an *ex situ* cuvette holder and *in situ* iSECCMS. It was reported that the excitation wavelength of 6CFL is at 490 nm (Prabhakaran et al., 2012). Correspondingly, a 495-nm laser was used as the excitation source in this experiment. For the *ex situ* cuvette measurement, 6CFL was dissolved in water. In the *in situ* measurement, 6CFL was immobilized in Nafion® as a thin film on the ITO electrode. The fluorescence of 6CFL in both measurements was observed between 515 and 650 nm (Figures 3B, 4A), which corroborates the accuracy and reliability of the probe and fluorescence spectroscopy apparatus used in the iSECCMS platform. The time-dependent stability of the 6CFL fluorescent probe was also analyzed. Previous studies indicate that 6CFL chemically degrades during photoexcitation, resulting in a gradual exponential decrease in fluorescence intensity over time (Song et al., 1996). However, substantial fluorescence decay was not observed during the *ex situ* cuvette experiment (Figure 3C). Because the *ex situ* experiment was conducted in bulk solution, it is likely that photo-degraded 6CFL molecules quickly diffuse out of the path of the laser beam, resulting in a steady replenishment of pristine 6CFL within the irradiated volume.

To characterize the stability of 6CFL fluorescence *in situ*, a capillary tip filled with and without 5% H_2_O_2_ solution in deionized (DI) water was brought into contact with the 6CFL embedded in a Nafion® film (without catalyst). Fluorescence was monitored using an inverted microscope (Figure 4A). Unlike the single broad fluorescent peak measured in the *ex situ* experiment, the *in situ* fluorescence spectrum of 6CFL instead shows three distinct peaks between 515 and 650 nm. These additional fluorescence peaks may be attributed to different solvent-coordinating environments of the 6CFL molecule, as well as a varying degree of local hydration in the polymer membrane. The intensity of the fluorescence peak at 580 nm was measured over time (Figure 4C). Even when the tip was filled with pure water, fluorescent decay was observed. This behavior may be attributed to continuous change in the local environment of the dye. In the *in situ* experiment, the 6CFL was embedded in a solid state Nafion® membrane where incomplete solvation around the 6CFL molecules may decrease the observed fluorescence. Additionally, prolonged exposure of the same relatively stationary 6CFL molecules to laser irradiation may degrade the dye without any chemically activated quenchers such as ROS. Therefore, the lower fluorescence and faster decay rates of 6CFL due to these factors were considered during our data analysis. To ensure that the iSECCMS platform is sensitive to the influence of ROS generation through fluorescence quenching, DI water was replaced with a 5% H_2_O_2_ solution. The resulting fluorescence demonstrated a greater rate of decay when compared to the pure water (Figure 4C). This observation indicates that the additional fluorescence decay is due to the presence of H_2_O_2_ and the generated ROS that decomposes the dye as expected. It also serves as an indicator of the sensitivity of the iSECCMS platform to peroxide generation during the ORR.

Next, we monitored the generation of H_2_O_2_/ROS on N-TaTiO_x_/C embedded in a 6CFL/Nafion® membrane. The fluorescence activity was measured using a 0.1 M H_2_SO_4_ solution purged with either Ar or O_2_. The theta capillary containing saturated electrolyte was brought into contact with the working electrode, and the fluorescence intensity was measured using the inverted microscope, as described above. The *in situ* LSV measurement on N-TaTiO_x_/C showed that the diffusion limited ORR occurred below ~0.3 V vs. RHE where the H_2_O_2_ generation rate is at a maximum (Figure 4B). Therefore, the fluorescence of 6CFL was monitored in both O_2_ and Ar-purged electrolyte after fixing the working potential at 0.3 V vs. NHE (Figure 4D). A slight increase in the fluorescence intensity was observed in the initial part of the Ar-saturated H_2_SO_4_ measurement presented in Figure 4D. This increase may be attributed to changes in the local hydration within the probing region of the optical microscope on the electrode. The fluorescence intensity of the 6CFL dye is very sensitive to such subtle changes in the local hydration, which may cause the initial increase in fluorescence intensity before settling down over longer times. Despite this anomaly, it is clear that the fluorescence decay of 6CFL in O_2_-saturated electrolyte is faster than in Ar-saturated electrolyte.

In order to obtain a quantitative estimate of the rate of ROS generation presented in Figures 4C,D, we used a similar rate equation to that previously reported (Prabhakaran et al., 2012) for the decay of 6CFL in the presence of ROS. To summarize, the decrease in fluorescence is given by

(1) $$\begin{array}{l} ROS+6CFL\to products\;\left(nonfluorescent\right) \end{array}$$

with a corresponding rate constant of

(2) $$\begin{array}{l} r_{1}=k_{1}C_{6CFL}C_{ROS} \end{array}$$

The reaction is assumed to be analogous to a batch reactor because 6CFL is initially charged, leading to the differential equation as follows (Prabhakaran et al., 2012):

(3) $$\begin{array}{l} \frac{dC_{6CFL}\left(t\right)}{dt}={-k}_{1}C_{6CFL}C_{ROS} \end{array}$$

Several simplifying assumptions are made to solve this equation. First, the concentration of ROS (*C*_*ROS*_) is treated as initially constant, giving a new constant *k*^*app*^ defined as

(4) $$\begin{array}{l} k^{app}=k_{1}C_{ROS} \end{array}$$

Second, the concentration of 6CFL is proportional to the intensity of fluorescence, which allows the normalized intensity to be substituted for normalized decay in *C*_6*CFL*_

(5) $$\begin{array}{l} \frac{C_{6CFL}\left(t\right)}{C_{6CFL},\left(t=0\right)}\approx \frac{I}{I_{0}} \end{array}$$

Substituting Equations (4, 5) into the differential equation [Equation (3)] and solving yields.

(6) $$\begin{array}{l} \ln\left(\frac{I}{I_{0}}\right)={-k}^{app}t \end{array}$$

**Figure 5:**
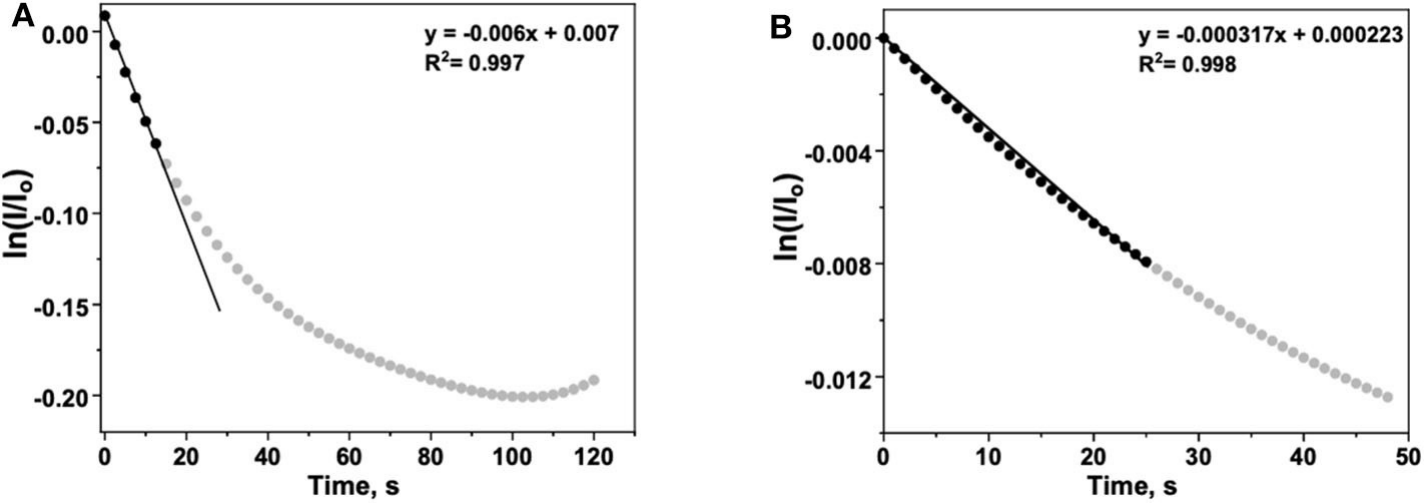
**(A)** Background-corrected time-dependent $\ln\left(\frac{I}{I_{0}}\right)$ for 6CFL fluorescence in the presence of an aqueous H_2_O_2_ solution. The initial rate is approximated by fitting the first several data points (*t* < 15 s) with Equation (6), a linear function with a slope of −*k*^*app*^. **(B)** Argon-subtracted time-dependent $\ln\left(\frac{I}{I_{0}}\right)$ for 6CFL fluorescence decay during the ORR with N-TaTiOx/C on ITO. The working electrode potential was maintained at 0.3 V vs. reversible hydrogen electrode (RHE). The initial rate is approximated by fitting the first several data points (*t* < 25 s) with Equation (6), a linear function with a slope of −*k*^*app*^.

Equation (6) provides a mathematical expression that can be applied to Figures 4C,D. In the case of Figure 4C, the decay in fluorescence results from both ROS generation and photodegradation. To isolate the effects of ROS generation, the time-dependent normalized intensity $\left(\frac{I}{I_{0}}\right)$ of a tip filled with water (no peroxide present) is subtracted from the analogous data for a tip with 5% H_2_O_2_. The ln of the “corrected” normalized intensity vs. t is plotted, with an initial slope equal to −*k*^*app*^ (Figure 5A).

(7) $$\begin{array}{l} k^{app}=k_{1}C_{ROS}=5.61\times 10^{-3}s^{-1} \end{array}$$

The apparent rate constant *k*^*app*^ can be interpreted as the rate constant for fluorescence decay described in Equation (1) and is analogous to the decay constant associated with ROS production (Prabhakaran et al., 2012).

In Figure 4D, the contribution of the ORR to fluorescence decay is measured by performing two similar experiments in which the electrolyte is purged with either argon or oxygen. The increased rate of decay in the latter is attributed to the presence of ROS that form as the oxygen dissolved in the electrolyte undergoes the incomplete ORR. To quantify the increase in fluorescence decay, the normalized intensity $\left(\frac{I}{I_{0}}\right)$ of fluorescence in the argon-purged experiment is subtracted from the fluorescence measured with an oxygen saturated electrolyte (Figure 5B). Performing an identical analysis, as described for Figure 5A, yields an apparent rate constant of

(8) $$\begin{array}{l} k^{app}=k_{1}C_{ROS}=3.17\times 10^{-4}s^{-1} \end{array}$$

This lower value reflects the initial rate of fluorescence decay resulting only from ROS generation during the ORR. The *k*^*app*^ determined for the electrode used with H_2_O_2_ is higher than that for the electrode used with O_2_, which confirms that the ROS generation is less in the latter case, as expected. Additionally, the values of *k*^*app*^ calculated herein are in close agreement with the values reported previously using *in situ* fluorescence measurements performed in an operating PEM fuel cell MEA (Prabhakaran et al., 2012). Based on our calculations and experimental observations, our results demonstrate that the 6CFL-based fluorescent probe used in the iSECCMS platform is sensitive toward localized ROS generation on ORR catalyst layers. Combining fluorescence microspectroscopy with the SECCM platform enabled us to understand the reaction kinetics and the origin of degradation at localized regions on the electrodes. While the functionality of the iSECCMS presented in this work was focused on the ORR which is relevant to PEFC cathodes, it can be expanded to study numerous electrochemical interfacial reactions in the field of energy conversion and storage as well as electrosynthesis where correlative monitoring of intermediates at localized regions is required.

## Conclusion

In this work, we report the development and versatile functionality of a new iSECCMS platform that enables correlated *in situ* electrochemical and spectroscopic measurements with high spatial resolution. The iSECCMS platform was used to study the *in situ* ROS/H_2_O_2_ generation rate on a potential Pt-free ORR catalyst, N-TiTaO_x_/C, as an example model system. The ORR activity measured with the iSECCMS platform is in agreement with *ex situ* RRDE measurements, which showed generation of the partial reduction intermediate, H_2_O_2_, on this catalyst. The iSECCMS platform was used to monitor the generation of ROS/H_2_O_2_ during the ORR on active sites of these catalysts with a spatial resolution of 10–20 μm. Future work will focus on improving the stability of the 6CFL probe and attaining higher lateral resolution. Furthermore, this platform will be used to study the effect of mitigation strategies, such as incorporating radical scavengers with the goal of eliminating the detrimental effects of ROS generation on TaTiO_x_/C catalysts, for which molecular-level integration of the ORR catalyst and radical scavenger is necessary. This integration will aid in the development of efficient and inexpensive ORR catalysts for PEFC. More broadly, the novel iSECCMS platform developed and evaluated herein may be used to study the interfacial spectrochemistry of photoelectrochemically active redox and catalytically active electrodes. The insights obtained from these experiments may guide the development of advanced electrochemical separation and energy storage technologies where local heterogeneity of electrodes plays an important role in controlling the performance of electrode–electrolyte interfaces.

## Data Availability Statement

The raw data supporting the conclusions of this article will be made available by the authors, without undue reservation.

## Author Contributions

JE performed experiments using the iSECCMS platform and helped write the paper. XX prepared the catalyst. YS designed and helped prepare the catalyst and interpret the data. PE-K assisted with building the spectroscopy component of the instrument. GJ helped interpret the data and write the paper. VP built the iSECCMS platform, oversaw the effort and wrote the paper. All authors contributed to the article and approved the submitted version.

## Conflict of Interest

The authors declare that the research was conducted in the absence of any commercial or financial relationships that could be construed as a potential conflict of interest.

## References

[B1] Baker L. A. (2018). Perspective and prospectus on single-entity electrochemistry. J. Am. Chem. Soc. 140, 15549–15559. 10.1021/jacs.8b09747 30388887 PMC8720287

[B2] Bentley C. L. Kang M. Unwin P. R. (2017). Scanning electrochemical cell microscopy: new perspectives on electrode processes in action. Curr. Opin. Electrochem. 6, 23–30. 10.1016/j.coelec.2017.06.011

[B3] Bosnjakovic A. Kadirov M. K. Schlick S. (2007). Using ESR spectroscopy to study radical intermediates in proton-exchange membranes exposed to oxygen radicals. Res. Chem. Intermed. 33:677–87. 10.1163/156856707782169372

[B4] Brasiliense V. Berto P. Combellas C. Tessier G. Kanoufi F. (2016). Electrochemistry of single nanodomains revealed by three-dimensional holographic microscopy. Acc. Chem. Res. 49, 2049–2057. 10.1021/acs.accounts.6b00335 27598333

[B5] Byers C. P. Hoener B. S. Chang W.-S. Yorulmaz M. Link S. Landes C. F. (2014a). Single-particle spectroscopy reveals heterogeneity in electrochemical tuning of the localized surface plasmon. J. Phys. Chem. B 118, 14047–14055. 10.1021/jp504454y 24971712 PMC4266331

[B6] Byers J. C. Güell A. G. Unwin P. R. (2014b). Nanoscale electrocatalysis: visualizing oxygen reduction at pristine, kinked, and oxidized sites on individual carbon nanotubes. J. Am. Chem. Soc. 136, 11252–11255. 10.1021/ja505708y 25061694 PMC4140457

[B7] Chirea M. Collins S. S. E. Wei X. Mulvaney P. (2014). Spectroelectrochemistry of silver deposition on single gold nanocrystals. J. Phys. Chem. Lett. 5, 4331–4335. 10.1021/jz502349x 26273983

[B8] Coleman E. J. Co A. C. (2014). Galvanic displacement of Pt on nanoporous copper: An alternative synthetic route for obtaining robust and reliable oxygen reduction activity. J. Catal. 316, 191–200. 10.1016/j.jcat.2014.05.012

[B9] Coleman E. J. Co A. C. (2015). The complex inhibiting role of surface oxide in the oxygen reduction reaction. ACS Catal. 5, 7299–7311. 10.1021/acscatal.5b02122

[B10] Coms F. D. (2008). The chemistry of fuel cell membrane chemical degradation. ECS Trans. 16, 235–255. 10.1149/1.2981859

[B11] Daviddi E. Gonos K. L. Colburn A. W. Bentley C. L. Unwin P. R. (2019). Scanning electrochemical cell microscopy (SECCM) chronopotentiometry: development and applications in electroanalysis and electrocatalysis. Anal. Chem. 91, 9229–9237. 10.1021/acs.analchem.9b02091 31251561

[B12] Debe M. K. (2012). Electrocatalyst approaches and challenges for automotive fuel cells. Nature 486, 43–51. 10.1038/nature11115 22678278

[B13] Du L. Prabhakaran V. Xie X. Park S. Wang Y. Shao Y. (2020). Low-PGM and PGM-free catalysts for proton exchange membrane fuel cells: stability challenges and material solutions. Adv. Mater. 10.1002/adma.201908232. [Epub ahead of print]. 32240570

[B14] Epting W. K. Litster S. (2012). Effects of an agglomerate size distribution on the PEFC agglomerate model. Int. J. Hydrogen Energy 37, 8505–8511. 10.1016/j.ijhydene.2012.02.099

[B15] Fang X. Shen P. K. Song S. Stergiopoulos V. Tsiakaras P. (2009). Degradation of perfluorinated sulfonic acid films: an *in-situ* infrared spectro-electrochemical study. Polym. Degrad. Stab. 94, 1707–1713. 10.1016/j.polymdegradstab.2009.06.015

[B16] Gubler L. Dockheer S. M. Koppenol W. H. (2011). Radical (HO∙, H∙ and HOO∙) formation and ionomer degradation in polymer electrolyte fuel cells. J. Electrochem. Soc. 158, B755–B769. 10.1149/1.358104021655568

[B17] Hess K. C. Epting W. K. Litster S. (2011). Spatially resolved, *in situ* potential measurements through porous electrodes as applied to fuel cells. Anal. Chem. 83, 9492–9498. 10.1021/ac202231y 22040011

[B18] Holt K. B. Bard A. J. Show Y. Swain G. M. (2004). Scanning electrochemical microscopy and conductive probe atomic force microscopy studies of hydrogen-terminated boron-doped diamond electrodes with different doping levels. J. Phys. Chem. B 108, 15117–15127. 10.1021/jp048222x

[B19] Houchins C. Kleen J. G. Spendelow S. J. Kopasz J. Peterson D. Garland L. N.. (2012). U.S. DOE progress towards developing low-cost, high performance, durable polymer electrolyte membranes for fuel cell applications. Membranes 2, 855–878. 10.3390/membranes2040855 24958432 PMC4021924

[B20] Hu L. Zhang M. Komini Babu S. Kongkanand A. Litster S. (2019). Ionic conductivity over metal/water interfaces in ionomer-free fuel cell electrodes. ChemElectroChem 6, 2659–2666. 10.1002/celc.201900124

[B21] Ishihara A. Shibata Y. Mitsushima S. Ota K. (2008). Partially oxidized tantalum carbonitrides as a new nonplatinum cathode for PEFC−1–. J. Electrochem. Soc. 155, B400–B406. 10.1149/1.2839606

[B22] Kai T. Zoski C. G. Bard A. J. (2018). Scanning electrochemical microscopy at the nanometer level. Chem. Commun. 54, 1934–1947. 10.1039/C7CC09777H 29383337

[B23] Kinoshita K. (1992). Electrochemical Oxygen Technology. John Hoboken, NJ: Wiley and Sons.

[B24] Kumar A. Ramani V. (2013). Ta0.3Ti0.7O2 electrocatalyst supports exhibit exceptional electrochemical stability. J. Electrochem. Soc. 160, F1207–F1215. 10.1149/2.038311jes

[B25] Lefèvre M. Proietti E. Jaouen F. Dodelet J.-P. (2009). Iron-based catalysts with improved oxygen reduction activity in polymer electrolyte fuel cells. Science 324, 71–74. 10.1126/science.1170051 19342583

[B26] Li J. Zhang H. Samarakoon W. Shan W. Cullen D. A. Karakalos S.. (2019). Thermally driven structure and performance evolution of atomically dispersed fen4 sites for oxygen reduction. Angew. Chem. Int. Ed. 58, 18971–18980. 10.1002/anie.201909312 31633848

[B27] Li W. Liu J. Zhao D. (2016). Mesoporous materials for energy conversion and storage devices. Nat. Rev. Mater. 1:16023 10.1038/natrevmats.2016.23

[B28] Litster S. McLean G. (2004). PEM fuel cell electrodes. J. Power Sources 130, 61–76. 10.1016/j.jpowsour.2003.12.055

[B29] Mayrhofer K. Blizanac B. Arenz M. Stamenkovic V. Ross P. Markovic N. (2005). The impact of geometric and surface electronic properties of Pt-catalysts on the particle size effect in electrocatalysis. J. Phys. Chem. B 109, 14433–14440. 10.1021/jp051735z 16852816

[B30] McKelvey K. Edwards M. A. Unwin P. R. (2010). Intermittent contact–scanning electrochemical microscopy (IC–SECM): a new approach for tip positioning and simultaneous imaging of interfacial topography and activity. Anal. Chem. 82, 6334–6337. 10.1021/ac101099e 20583818

[B31] Mittal V. O. Russell Kunz H. Fenton J. M. (2006). Is H2O2 involved in the membrane degradation mechanism in PEMFC? Electrochem. Solid-State Lett. 9, A299–A302. 10.1149/1.2192696

[B32] Murakami Y. Kenji E. Nosaka A. Y. Nosaka Y. (2006). Direct detection of OH radicals diffused to the gas phase from the UV-irradiated photocatalytic TiO2 surfaces by means of laser-induced fluorescence spectroscopy. J. Phys. Chem. B 110, 16808–16811. 10.1021/jp063293c 16927965

[B33] Nesselberger M. Roefzaad M. Hamou R. F. Biedermann P. U. Schweinberger F. F. Kunz S.. (2013). The effect of particle proximity on the oxygen reduction rate of size-selected platinum clusters. Nat. Mater. 12, 919–924. 10.1038/nmat3712 23872730

[B34] Panchenko A. Dilger H. Kerres J. Hein M. Ullrich A. Kaz T. (2004a). *in-situ* spin trap electron paramagnetic resonance study of fuel cell processes. Phys. Chem. Chem. Phys. 6, 2891–2894. 10.1039/b404253k

[B35] Panchenko A. Dilger H. Möller E. Sixt T. Roduner E. (2004b). *In situ* EPR investigation of polymer electrolyte membrane degradation in fuel cell applications. J. Power Sources 127, 325–330. 10.1016/j.jpowsour.2003.09.047

[B36] Prabhakaran V. Arges C. G. Ramani V. (2012). Investigation of polymer electrolyte membrane chemical degradation and degradation mitigation using *in situ* fluorescence spectroscopy. Proc. Natl. Acad. Sci. U.S.A. 109, 1029–1034. 10.1073/pnas.1114672109 22219367 PMC3268320

[B37] Prabhakaran V. Arges C. G. Ramani V. (2013). *In situ* fluorescence spectroscopy correlates ionomer degradation to reactive oxygen species generation in an operating fuel cell. Phys. Chem. Chem. Phys. 15, 18965–18972. 10.1039/c3cp53919a 24092495

[B38] Prabhakaran V. Ramani V. (2014). Structurally-tuned nitrogen-doped cerium oxide exhibits exceptional regenerative free radical scavenging activity in polymer electrolytes. J. Electrochem. Soc. 161, F1–F9. 10.1149/2.004401jes

[B39] Ramesha G. K. Sampath S. (2009). Electrochemical reduction of oriented graphene oxide films: an *in situ* raman spectroelectrochemical study. J. Phys. Chem. C 113, 7985–7989. 10.1021/jp811377n

[B40] Sannomiya T. Dermutz H. Hafner C. Vörös J. Dahlin A. B. (2010). Electrochemistry on a localized surface plasmon resonance sensor. Langmuir 26, 7619–7626. 10.1021/la9042342 20020724

[B41] Song L. Varma C. A. Verhoeven J. W. Tanke H. J. (1996). Influence of the triplet excited state on the photobleaching kinetics of fluorescein in microscopy. Biophys. J. 70, 2959–2968. 10.1016/S0006-3495(96)79866-1 8744334 PMC1225276

[B42] Su P. Prabhakaran V. Johnson G. E. Laskin J. (2018). *In situ* infrared spectroelectrochemistry for understanding structural transformations of precisely defined ions at electrochemical interfaces. Anal. Chem. 90, 10935–10942. 10.1021/acs.analchem.8b02440 30130959

[B43] Sui S. Wang X. Zhou X. Su Y. Riffat S. Liu C.-J. (2017). A comprehensive review of Pt electrocatalysts for the oxygen reduction reaction: nanostructure, activity, mechanism and carbon support in PEM fuel cells. J. Mater. Chem. A 5, 1808–1825. 10.1039/C6TA08580F

[B44] Sun T. Tian B. Lu J. Su C. (2017). Recent advances in Fe (or Co)/N/C electrocatalysts for the oxygen reduction reaction in polymer electrolyte membrane fuel cells. J. Mater. Chem. A 5, 18933–18950. 10.1039/C7TA04915C

[B45] Trogadas P. Coppens M.-O. (2020). Nature-inspired electrocatalysts and devices for energy conversion. Chem. Soc. Rev. 49, 3107–3141. 10.1039/C8CS00797G 32239067

[B46] Trogadas P. Parrondo J. Mijangos F. Ramani V. (2011). Degradation mitigation in PEM fuel cells using metal nanoparticle additives. J. Mater. Chem. 21, 19381–19388. 10.1039/c1jm14077a

[B47] Wang W. Tao N. (2014). Detection, counting, and imaging of single nanoparticles. Anal. Chem. 86, 2–14. 10.1021/ac403890n 24328222 PMC4272604

[B48] Wang X. X. Prabhakaran V. He Y. Shao Y. Wu G. (2019). Iron-free cathode catalysts for proton-exchange-membrane fuel cells: cobalt catalysts and the peroxide mitigation approach. Adv. Mater. 31:1805126. 10.1002/adma.201805126 30706548

[B49] Wang Y. Shan X. Tao N. (2016). Emerging tools for studying single entity electrochemistry. Faraday Discuss. 193, 9–39. 10.1039/C6FD00180G 27722354

[B50] Wordsworth J. Benedetti T. M. Alinezhad A. Tilley R. D. Edwards M. A. Schuhmann W. (2020). The importance of nanoscale confinement to electrocatalytic performance. Chem. Sci. 11, 1233–1240. 10.1039/C9SC05611D PMC8148078 34123247

[B51] Wu D.-Y. Li J.-F. Ren B. Tian Z.-Q. (2008). Electrochemical surface-enhanced Raman spectroscopy of nanostructures. Chem. Soc. Rev. 37, 1025–1041. 10.1039/b707872m 18443687

[B52] Zhang H. Hwang S. Wang M. Feng Z. Karakalos S. Luo L.. (2017). Single atomic iron catalysts for oxygen reduction in acidic media: particle size control and thermal activation. J. Am. Chem. Soc. 139, 14143–14149. 10.1021/jacs.7b06514 28901758

[B53] Zhang Z.-W. Peng H.-J. Zhao M. Huang J.-Q. (2018). Heterogeneous/Homogeneous mediators for high-energy-density lithium–sulfur batteries: progress and prospects. Adv. Funct. Mater. 28:1707536 10.1002/adfm.201707536

[B54] Zhu Y. P. Guo C. Zheng Y. Qiao S.-Z. (2017). Surface and interface engineering of noble-metal-free electrocatalysts for efficient energy conversion processes. Acc. Chem. Res. 50, 915–923. 10.1021/acs.accounts.6b00635 28205437

